# Supplementary material for: Ultraviolet irradiation-responsive dynamic ultralong organic phosphorescence in polymeric systems
Source: Nat Commun. 2021 Apr 16;12:2297. doi: 10.1038/s41467-021-22609-y (PMC8052444; doi:10.1038/s41467-021-22609-y)
Supplement: Supplementary file 1 — Supplementary Information [file 41467_2021_22609_MOESM1_ESM.pdf]

Supplementary Information

Ultraviolet irradiation-responsive dynamic ultralong organic phosphorescence in polymeric systems

Zhang et al.

Supplementary Methods

Materials

The regents used in experiments including (1,1'-biphenyl)-2,2'-dicarboxaldehyde (2,2-DB, 97%), (1,1'-biphenyl)-4,4'-dicarboxaldehyde (4,4-DB, 98%), bisphenol A (ABP, 99%), diphenyl sulfone (DP, 99%), 4,4'-oxydibenzaldehyde (BFPE, 98%), 4,4'-oxydiphenol (ODP, 98%), 4,4'-sulfonyldiphenol (SDP, 99%), and 4,4'-thiodiphenol (TDP, 99%), they were purchased from EnergyChemical, Tokyo Chemical Industry (TCI), and Sigma-Aldrich. Polyvinyl alcohol (PVA) (molecular weight = 85,000 to 124,000 g/mol, 100 % hydrolyzed) was purchased from Sigma-Aldrich. Unless otherwise noted, all chemicals were used without further purification.

Measurements and Methods

The samples were measured in the film state. All films were irradiated by UV-254 nm lamp. FTIR spectra were recorded using a Nicolet iS-10 Fourier Transform Infrared Spectrometer. Thermogravimetric analysis (TGA) was carried out under nitrogen atmosphere from 30 to 800 ºC, at a heating rate of 10 ºC / min. Differential scanning calorimetry (DSC) was conducted on TA Q20 at a heating rate of 10 ºC / min under nitrogen. UV-vis absorption spectra were recorded on a Shimadzu UV-3600 UV-Vis-NIR spectrophotometer. The lifetime, time-resolved excitation spectra, steady-state and time-resolved emission spectra, and temperature-dependent photoluminescence spectra were measured on a Edinburgh FLS 1000 fluorescence spectrophotometer equipped with a xenon lamp (450 W), laser device (280 nm, 365 nm), or a microsecond flash-lamp (100 W). The photoluminescence quantum efficiency was determined on a Edinburgh FLS 1000 fluorescence spectrophotometer equipped with an integrating sphere. The lifetime (τ) of the luminescence was obtained by fitting the decay curve with a multi-exponential decay function (1):

$$I(\mathrm{t}) = \sum_{\mathrm{i}} A_{\mathrm{i}} \mathrm{e}^{-\frac{\mathrm{t}}{\tau_{\mathrm{i}}}} \qquad (1)$$

where A_i and τ_i represent the amplitudes and lifetimes of the individual components for multi-exponential decay profiles, respectively. Powder X-ray diffraction (XRD)

measurements were recorded on a Rigaku D/Max-2500 using Cu Kα radiation with 2θ range of 5 - 50°, 40 KeV, and 30 mA having a scanning rate of 0.01° s^{-1} (2θ) at room temperature. Scanning electron microscope (SEM) images were performed on a JSM - 6700F (JEOL). The luminous intensity was measured at PR-305 detector (Excitation time, 10 s; Steady-state interval, 1 s; Scan time, 2 min). EPR spectra were recorded on an A300 - 10 / 12 ESR spectrometer (300 K, film state). The photos and supporting videos were recorded by a Canon EOS 80D camera. For the theoretical simulations, the geometry of the ground state in the isolated state was fully optimized with density functional theory (DFT) with B3LYP hybrid functional at the basis set levelof 6-31G**. All the excited state geometries were optimized by the time-dependent DFT (TD-DFT) with B3LYP functional at the same basis set level as the ground state. The excitation energy in the n-th singlet (S_n) and n-th triplet (T_n) states of monomer and multimer was obtained by TD-DFT calculations on the M062X/6-31G(d,p) level of theory. All the above calculations were performed using Gaussian 09 in the PowerLeader workstation.[1]

Kinetic calculations and photophysical rate constants

Based on the measured fluorescence and phosphorescence quantum yield (PLQY) efficiency and emission lifetime, the radiative and nonradiative decay rates and intersystem crossing (ISC) rate can be calculated according to standard photophysical dynamic equations.[2-4] Theoretically, the lifetime and radiative PLQY of fluorescence (τ_F and Φ_F) and phosphorescence (τ_P and Φ_P), including PLQY of ISC (Φ_{ISC}), can be expressed in equations (2-5), respectively.

$$\tau_{Fluo.} = \frac{1}{K_r^{Fluo.} + K_{nr}^{Fluo.} + K_{ISC}} \quad (2)$$

$$\tau_{Phos.} = \frac{1}{K_r^{Phos.} + K_{nr}^{Phos.}} \quad (3)$$

$$\phi_{Fluo.} = \frac{K_r^{Fluo.}}{K_r^{Fluo.} + K_{nr}^{Fluo.} + K_{ISC}} = K_r^{Fluo.} \times \tau_{Fluo.} \quad (4)$$

$$\phi_{ISC} = \frac{K_{ISC}}{K_r^{Fluo.} + K_{nr}^{Fluo.} + K_{ISC}} = K_{ISC} \times \tau_{Fluo.} \quad (5)$$

where $K_r^{Fluo.}$ and $K_{nr}^{Fluo.}$ represent the radiative and nonradiative rate constant of fluorescence

respectively, K_{ISC} and ϕ_{ISC} are the rate constant and PLQY of ISC respectively, $K_{\mathrm{r}}^{Phos.}$ and $K_{\mathrm{nr}}^{\mathrm{Phos.}}$ are the radiative and nonradiative rate constant of room-temperature phosphorescence respectively. The essential assumption is the nonradiative rate constant of the triplet ($K_{\mathrm{nr}}^{Phos.}$) is much lower than $K_{\mathrm{nr}}^{Phos.}$, owing to the fact that the triplet excitons stabilized by UV-254 nm irradiation are stable enough. Combined with the above equations, K_{ISC} and $K_{\mathrm{nr}}^{Phos.}$ can be expressed in equations (8) and (10).

$$K_{\mathrm{r}}^{Fluo.} = \frac{\phi_{Fluo.}}{\tau_{Fluo.}} \qquad (6)$$

$$K_{\mathrm{nr}}^{\mathrm{Fluo.}} = \frac{(1 - \phi_{\mathrm{Fluo.}} - \phi_{\mathrm{phos.}})}{\tau_{\mathrm{Fluo.}}} \qquad (7)$$

$$K_{ISC} = \frac{\phi_{\mathrm{Phos.}}}{\tau_{\mathrm{Fluo.}}} \qquad (8)$$

$$K_{\mathrm{r}}^{\mathrm{Phos.}} = \frac{\phi_{\mathrm{Phos.}}}{\tau_{Phos.}} \qquad (9)$$

$$K_{\mathrm{nr}}^{Phos.} = \frac{(1 - \phi_{\mathrm{phos.}})}{\tau_{\mathrm{Phos.}}} \qquad (10)$$

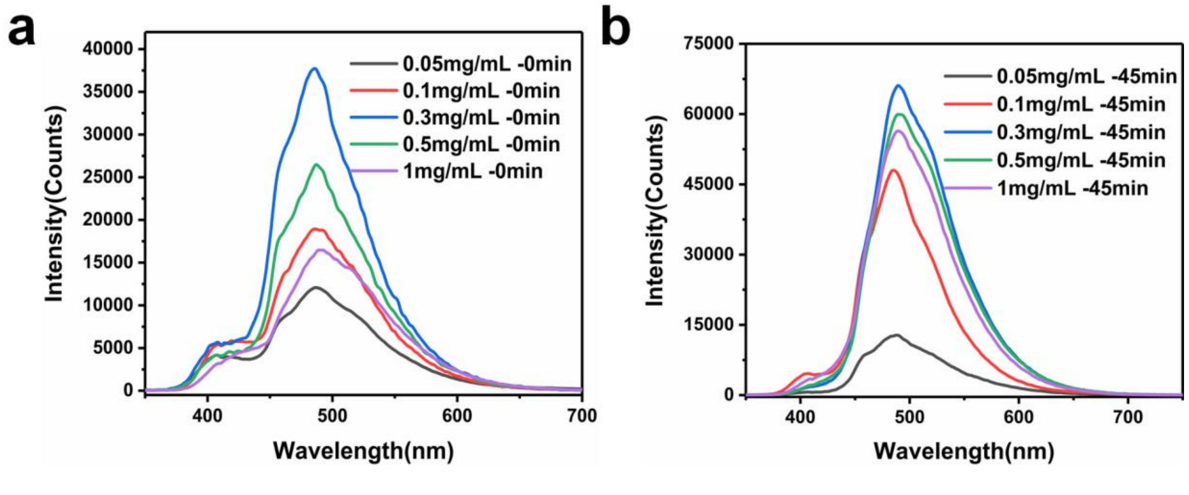

Supplementary Figure 1. Delayed photoluminescence spectral studies. Delayed photoluminescence spectra of SDP doped film at different doping concentrations (a) without irradiation and (b) with 45 min continuous irradiation.

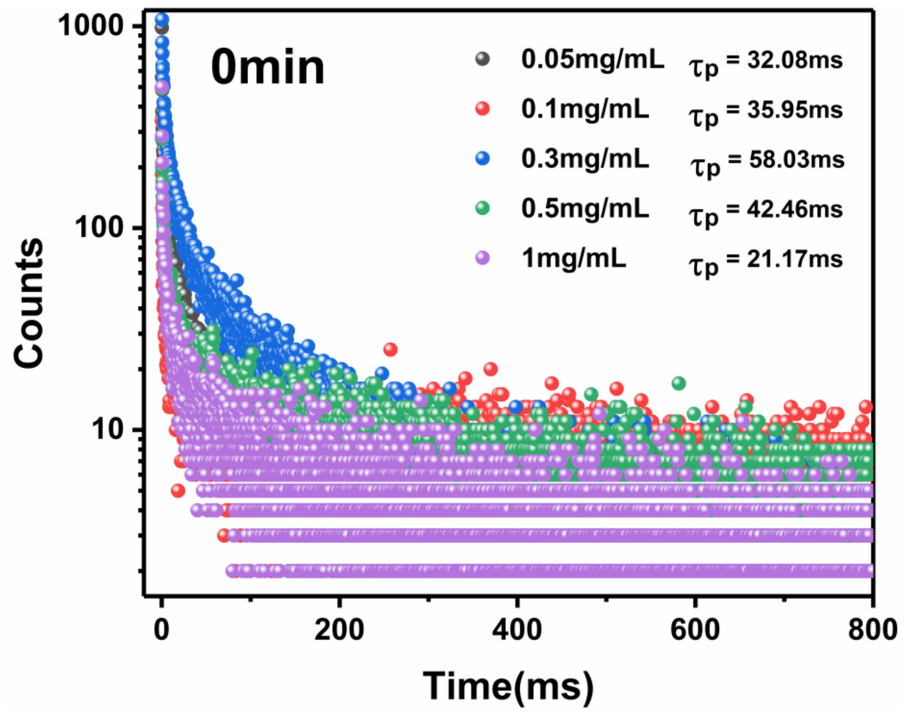

Supplementary Figure 2. Lifetime decay profiles of the emission band around 488 nm for SDP doped films at different doping concentrations without the irradiation.

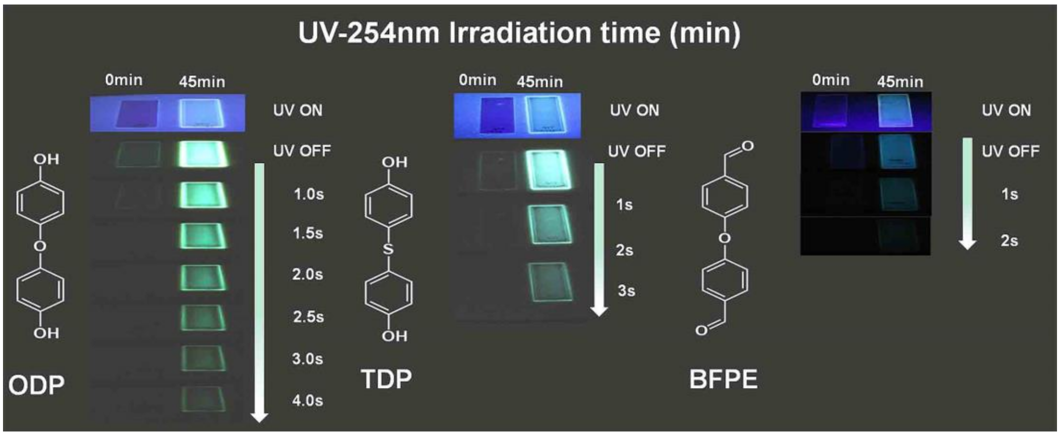

Supplementary Figure 3. Comparison of the phosphorescence emission among ODP, TDP, and BFPE doped films at 0.3 mg/mL doping concentration. They were observed at different time intervals before and after switching off light excitation at ambient conditions.

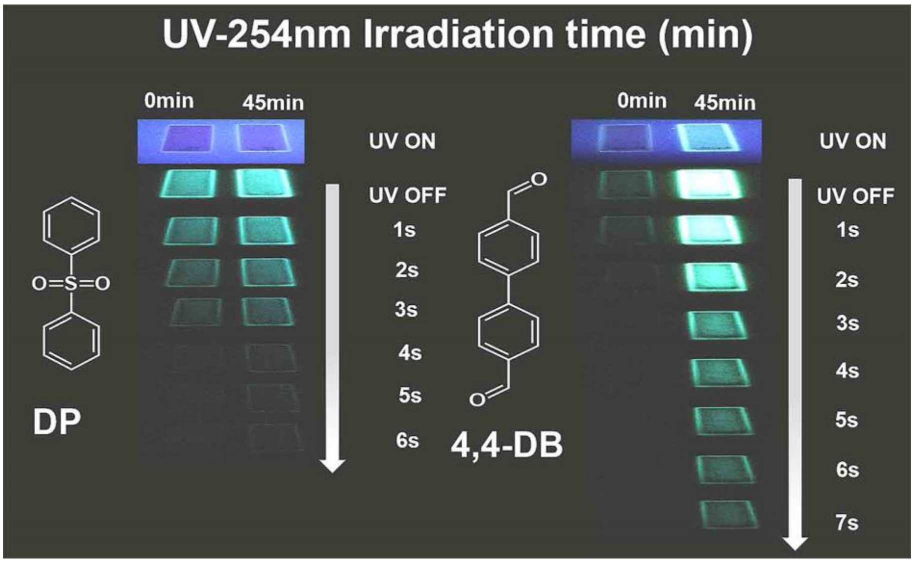

Supplementary Figure 4. Comparison of the phosphorescence emission for DP and 4,4-DB doped films at 0.3 mg/mL doping concentration. They were observed at different time intervals before and after switching off light excitation at ambient conditions.

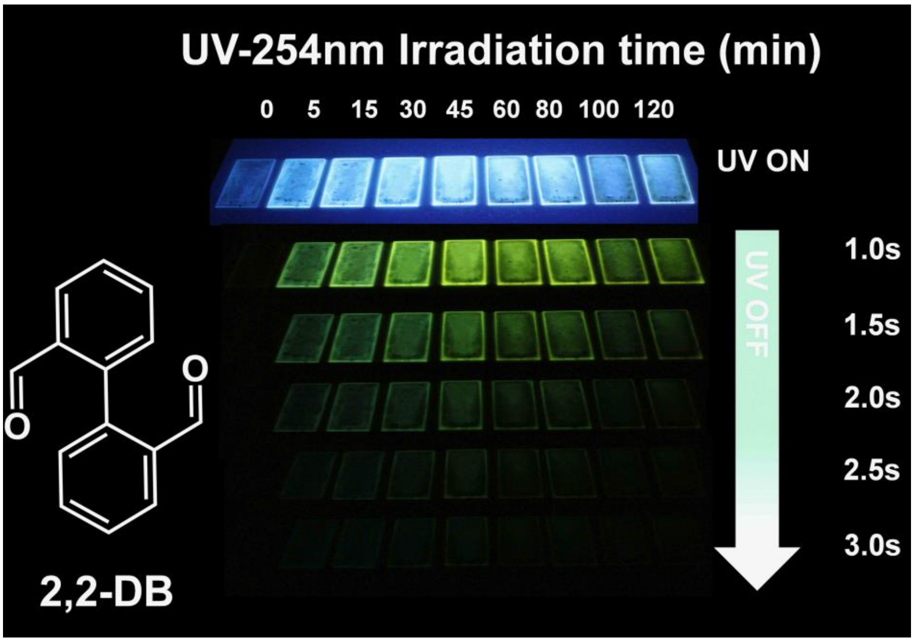

Supplementary Figure 5. Comparison of the phosphorescence emission for 2,2-DB doped film at 0.3 mg/mL doping concentration. It was observed at different time intervals before and after switching off light excitation at ambient conditions.

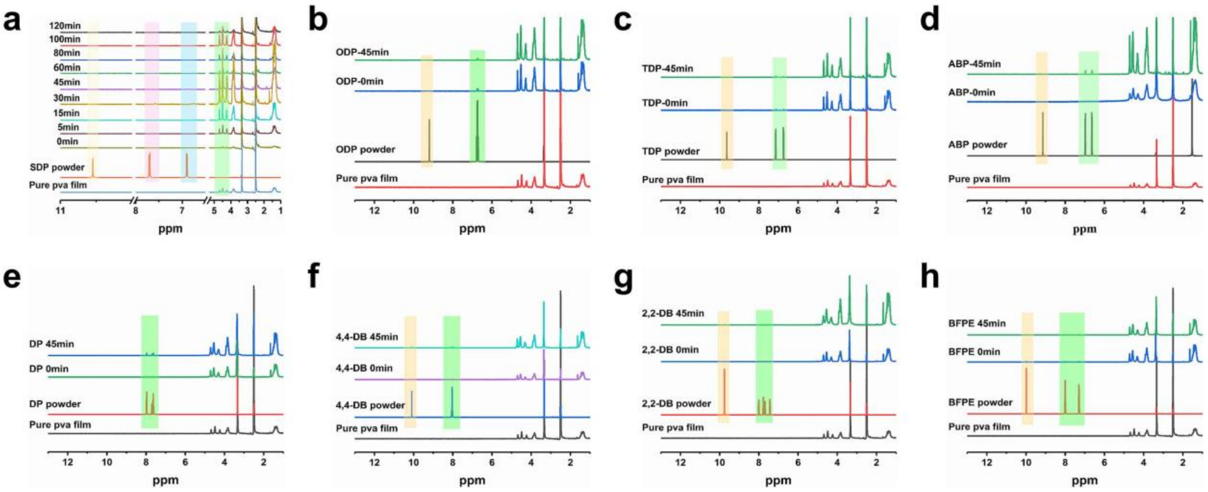

Supplementary Figure 6. ^{1}H NMR spectral studies. ^{1}H NMR spectra of (a) SDP, (b) ODP, (c) TDP, (d) ABP, (e) DP, (f) 4,4-DB, (g) 2,2-DP, and (h) BFPE doped films at 0.3 mg/mL doping concentration in DMSO-d_6. SDP-based film was irradiated from 0 min to 120 min, and others were irradiated for 45 min.

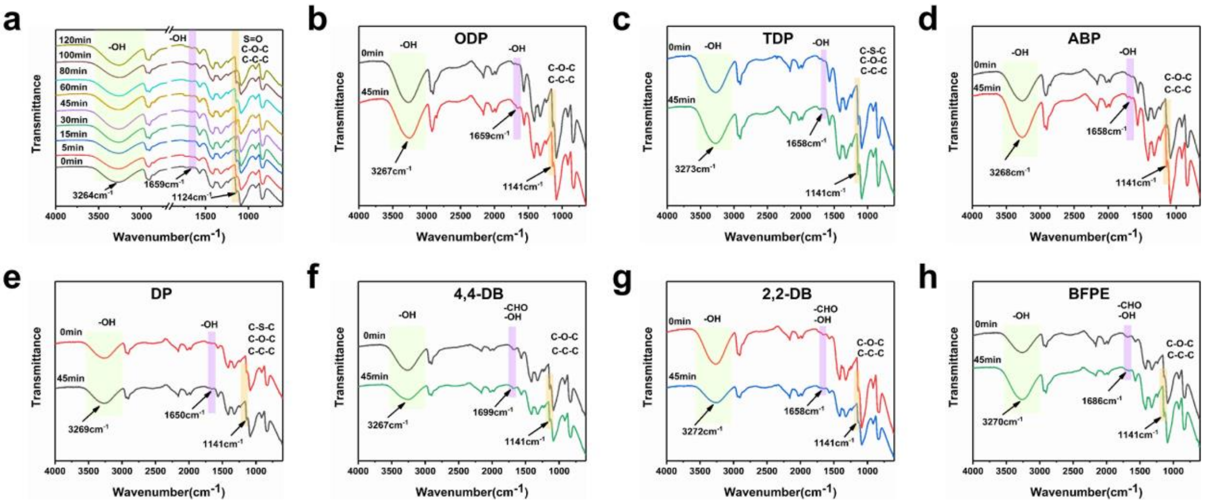

Supplementary Figure 7. FT-TR spectral studies. FT-TR spectra of (a) SDP, (b) ODP, (c) TDP, (d) ABP, (e) DP, (f) 4,4-DB, (g) 2,2-DP, and (h) BFPE doped films at 0.3 mg/mL doping concentration. These films were irradiated at different times.

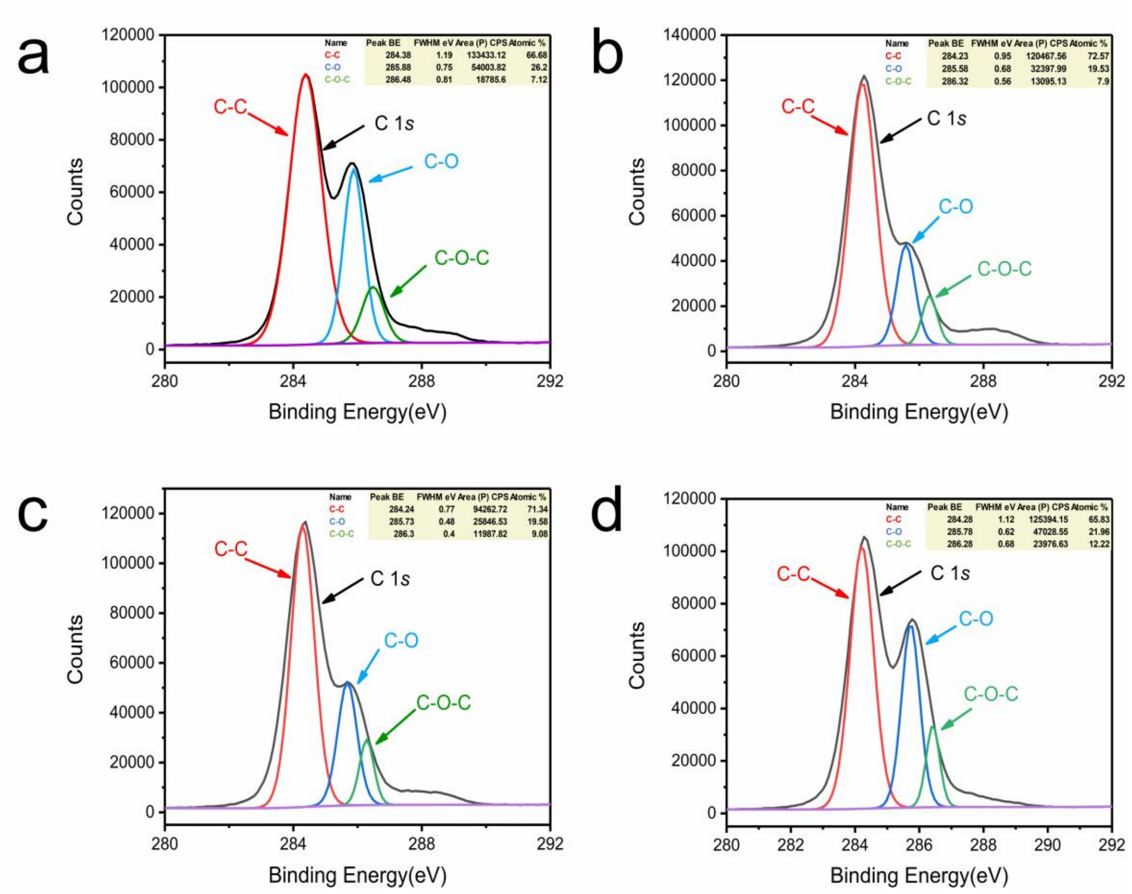

Supplementary Figure 8. High-resolution XPS spectral studies. High-resolution XPS spectra of SDP doped film at 0.3 mg/mL doping concentration under irradiation for (a) 0 min, (b) 5 min, (c) 45 min, and (d) 120 min.

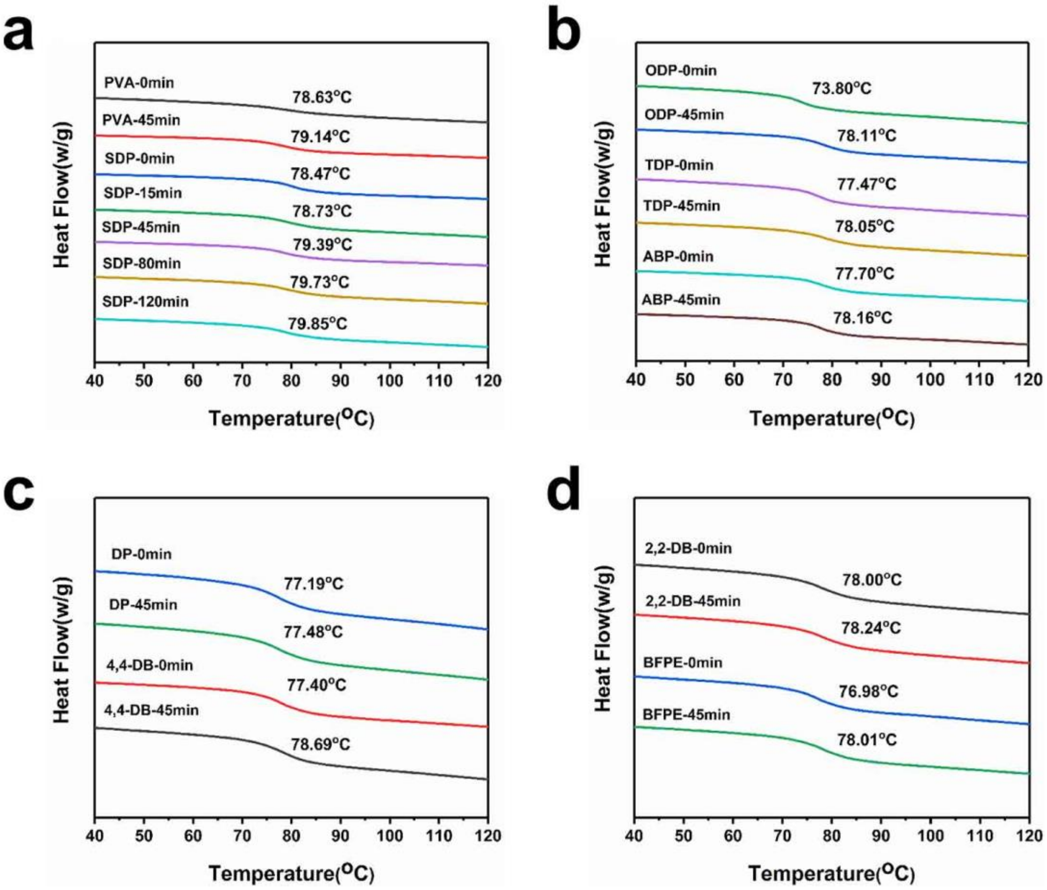

Supplementary Figure 9. DSC studies. (a-d) DSC curves of SDP, ODP, TDP, ABP, DP, 4,4-DB, 2,2-DP, and BFPE doped films at 0.3 mg/mL doping concentration under different irradiation times.

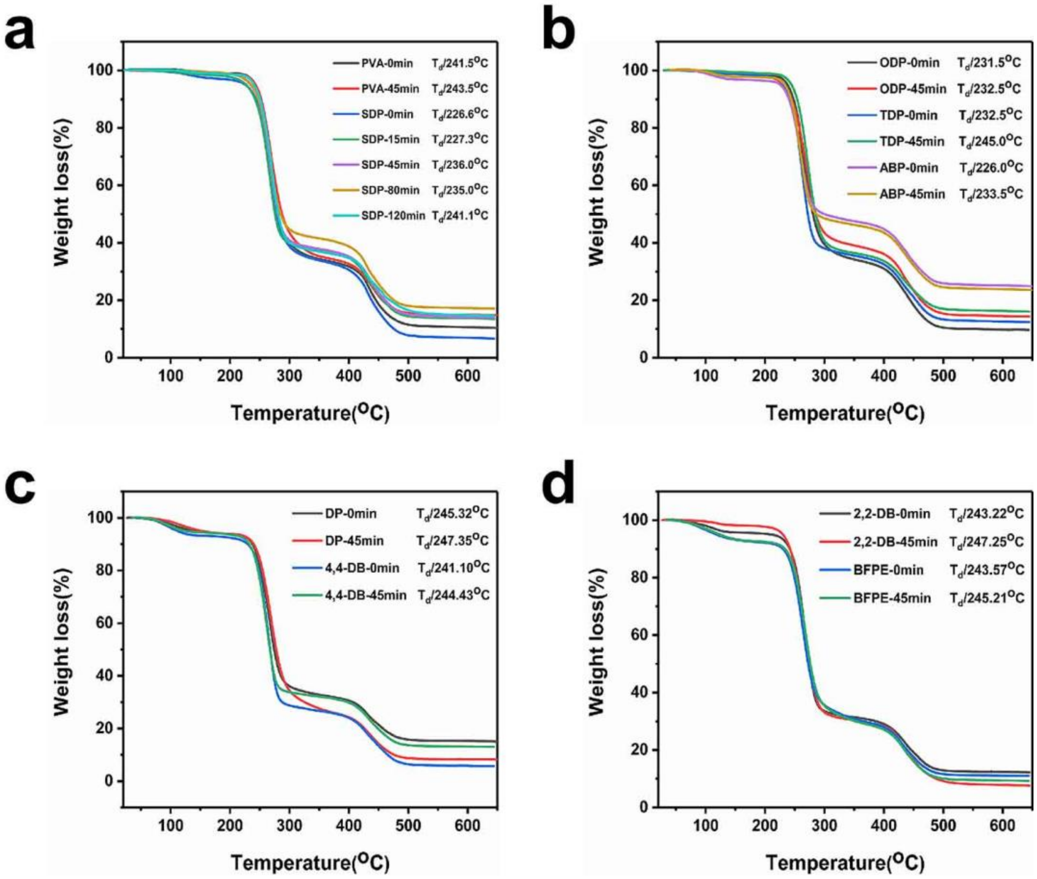

Supplementary Figure 10. TGA studies. (a-d) TGA curves of SDP, ODP, TDP, ABP, DP, 4,4-DB, 2,2-DP, and BFPE doped films at 0.3 mg/mL doping concentration with different irradiation times.

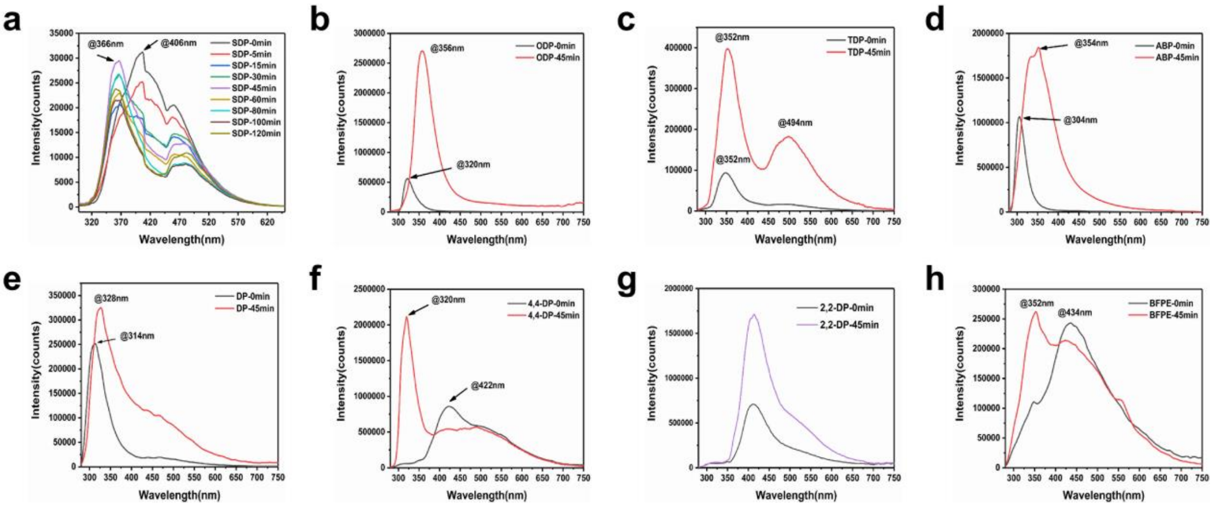

Supplementary Figure 11. Photoluminescence spectral studies. Photoluminescence spectra of (a) SDP, (b) ODP, (c) TDP, (d) ABP, (e) DP, (f) 4,4-DB, (g) 2,2-DP, and (h) BFPE doped films at 0.3 mg/mL doping concentration with different irradiation times at room temperature.

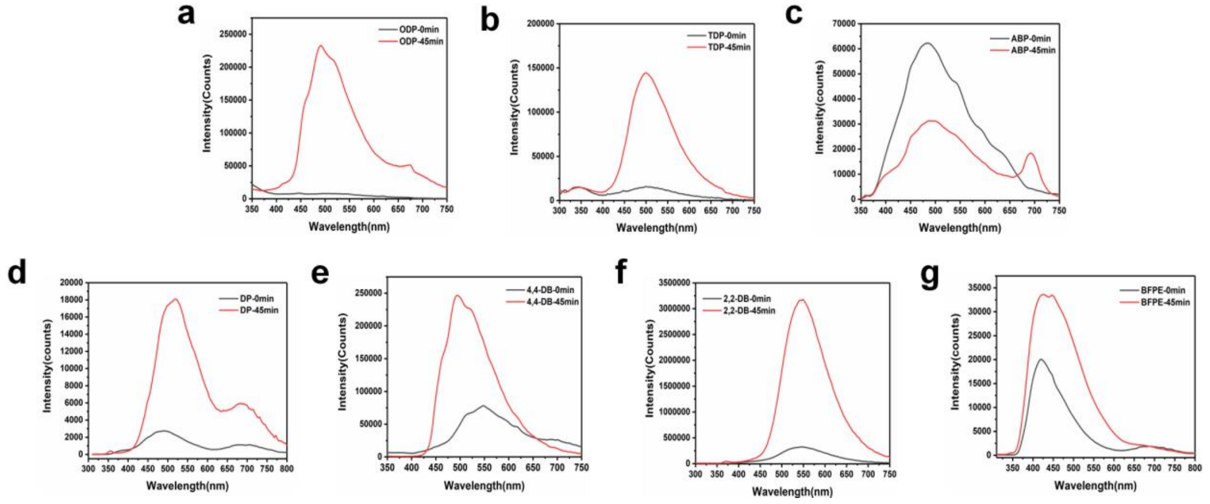

Supplementary Figure 12. Delayed photoluminescence spectral studies. Delayed photoluminescence spectra of (a) ODP, (b) TDP, (c) ABP, (d) DP, (e) 4,4-DB, (f) 2,2-DP, and (g) BFPE doped films at 0.3 mg/mL doping concentration with 0 min and 45 min irradiation at room temperature.

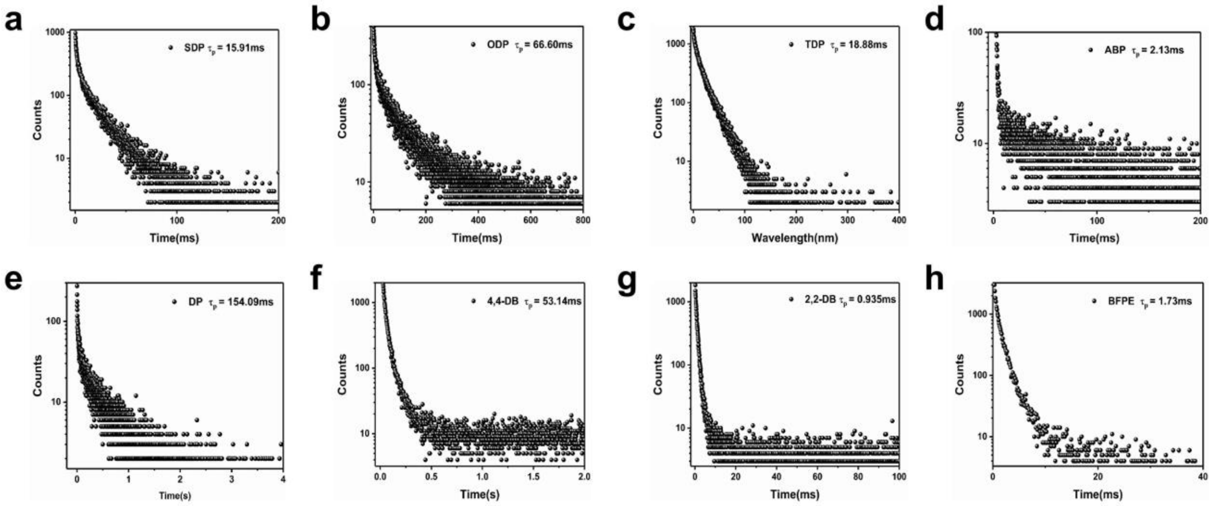

Supplementary Figure 13. Phosphorescence decay studies. Phosphorescence decay curves of (a) SDP, (b) ODP, (c) TDP, (d) ABP, (e) DP, (f) 4,4-DB, (g) 2,2-DP, and (h) BFPE at power state.

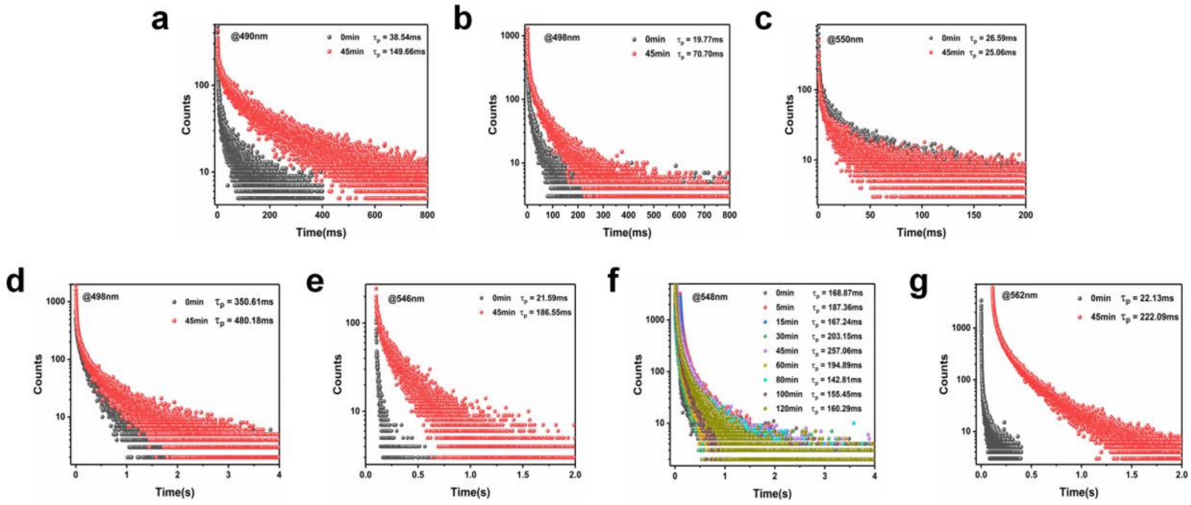

Supplementary Figure 14. Phosphorescence decay studies. Phosphorescence decay curves of (a) ODP, (b) TDP, (c) ABP, (d) DP, (e) 4,4-DB, (f) 2,2-DP, and (g) BFPE doped films at 0.3 mg/mL doping concentration with 0 min and 45 min irradiation at room temperature.

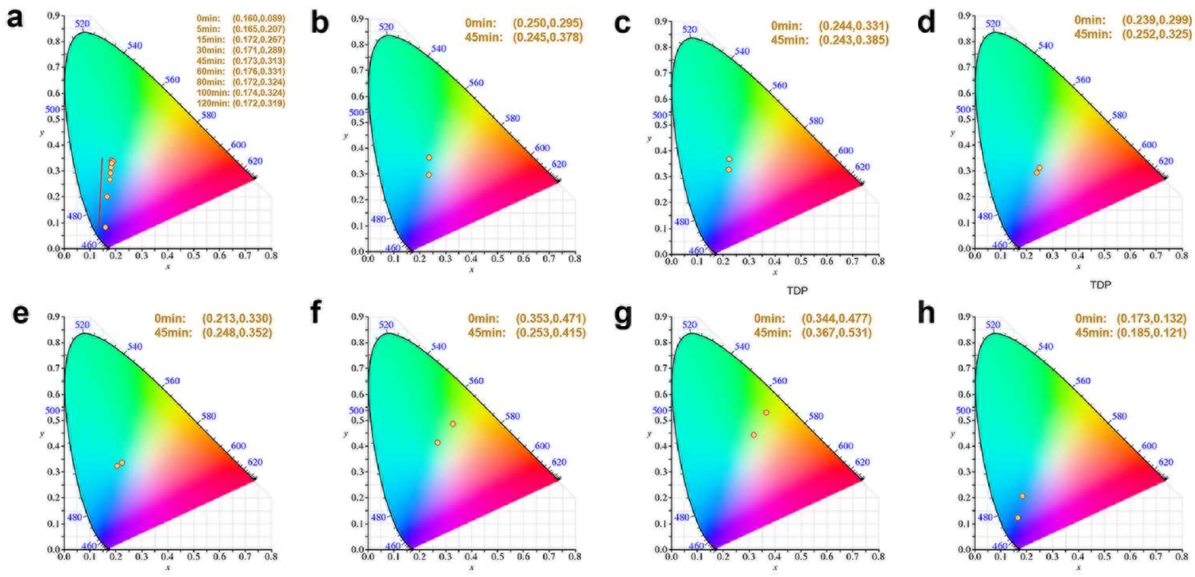

Supplementary Figure 15. Trajectory of color modulation. Trajectory of color modulation recorded from (a) SDP, (b) ODP, (c) TDP, (d) ABP, (e) DP, (f) 4,4-DB, (g) 2,2-DP, and (h) BFPE doped films at 0.3 mg/mL doping concentration after 0 min and 45 min irradiation at room temperature.

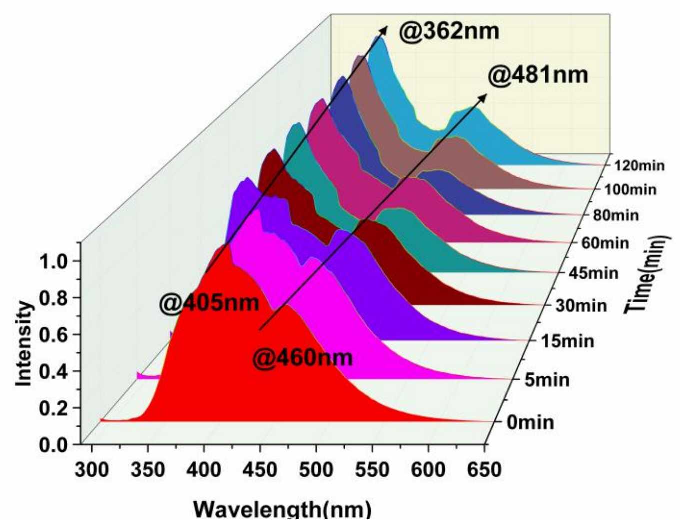

Supplementary Figure 16. Normalized photoluminescence spectra of SDP doped film at 0.3 mg/mL doping concentration with different irradiation times at room temperature.

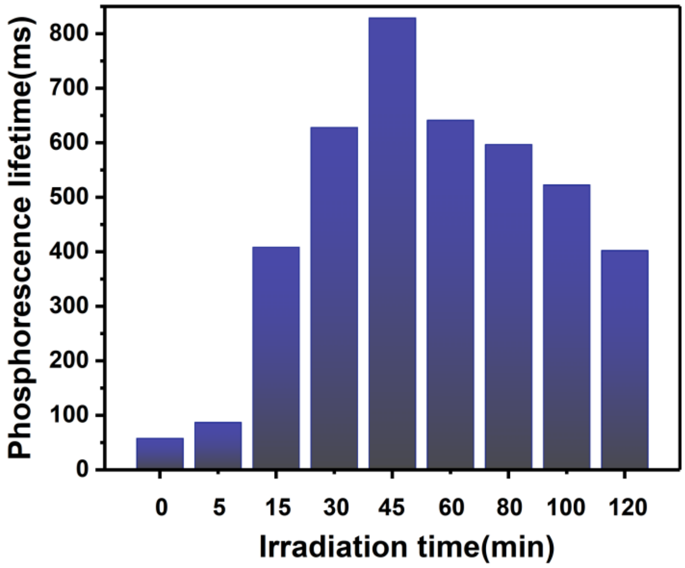

Supplementary Figure 17. Comparison of phosphorescent lifetime for SDP doped film at 0.3 mg/mL doping concentration with different irradiation times at room temperature.

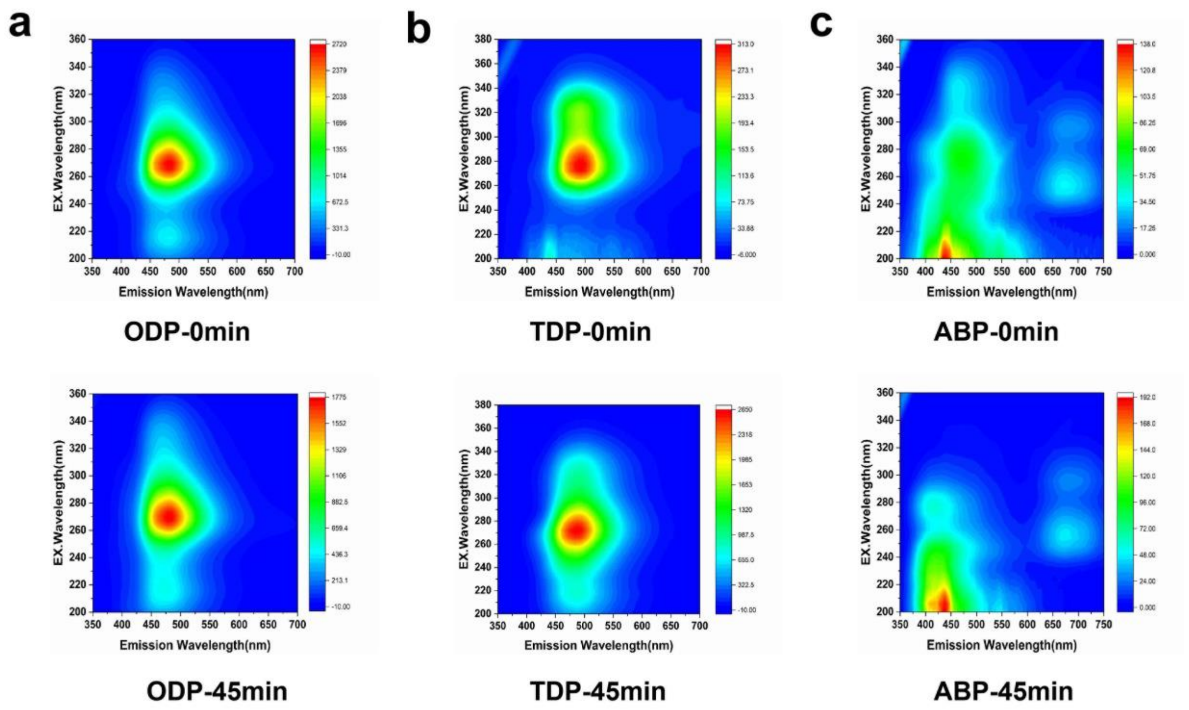

Supplementary Figure 18. Phosphorescence-excitation mapping studies. Phosphorescence-excitation mapping of (a) ODP, (b) TDP, and (c) ABP doped films with 0 min and 45 min irradiation under ambient conditions.

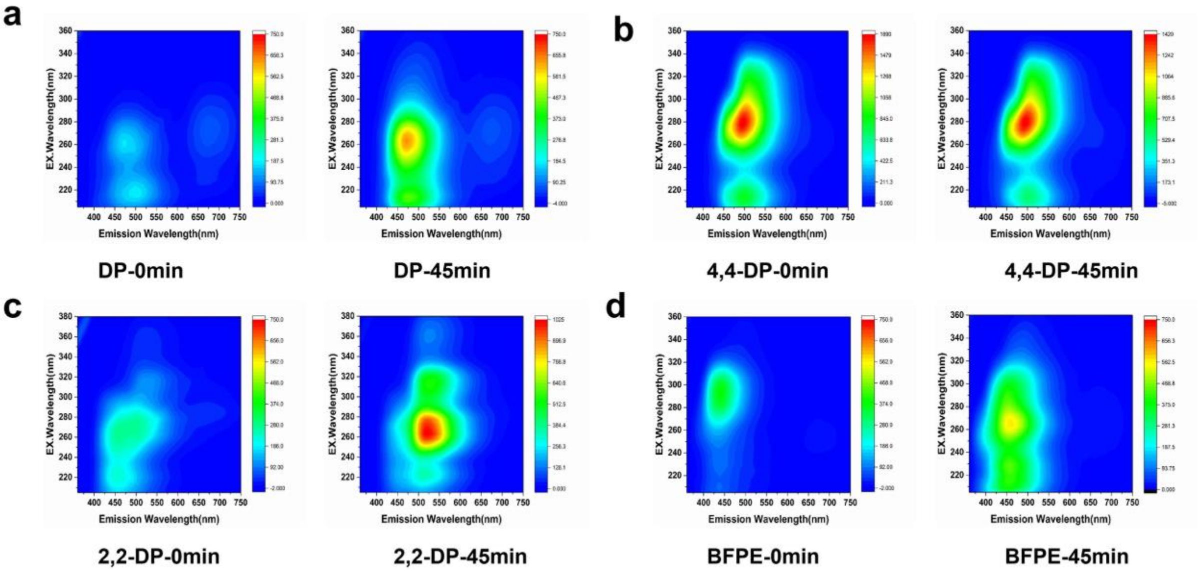

Supplementary Figure 19. Phosphorescence-excitation mapping studies. Phosphorescence-excitation mapping of (a) DP, (b) 4,4-DB, (c) 2,2-DB, and (d) BFPE doped films with 0 min and 45 min irradiation under ambient conditions.

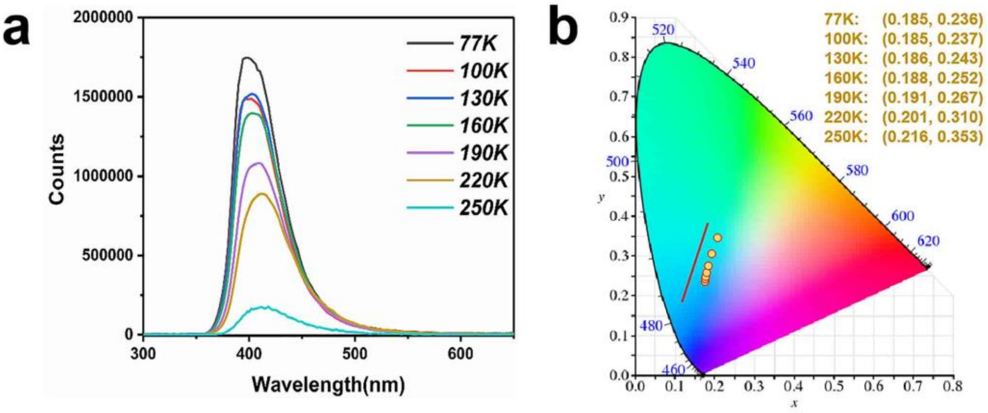

Supplementary Figure 20. Temperature-dependent delayed photoluminescence spectra and trajectory of color modulation. (a) Temperature-dependent delayed photoluminescence spectra of SDP doped film with 0.3 mg/mL doping concentration from 77 K to 250 K without irradiation. (b) Trajectory of color modulation for photoluminescence spectra in Figure 3e with 45 min irradiation, corresponding to the ICE coordinate.

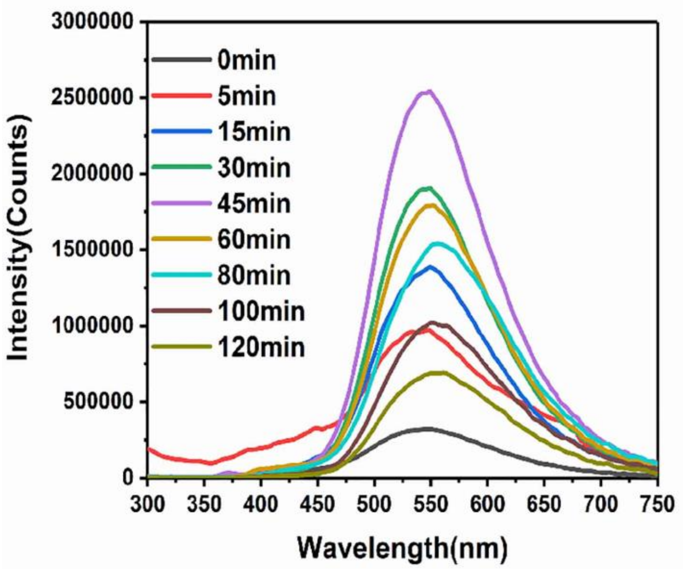

Supplementary Figure 21. Delayed photoluminescence spectra of 2,2-DB doped film with 0.3 mg/mL doping concentration under irradiation from 0 min to 120 min at room temperature.

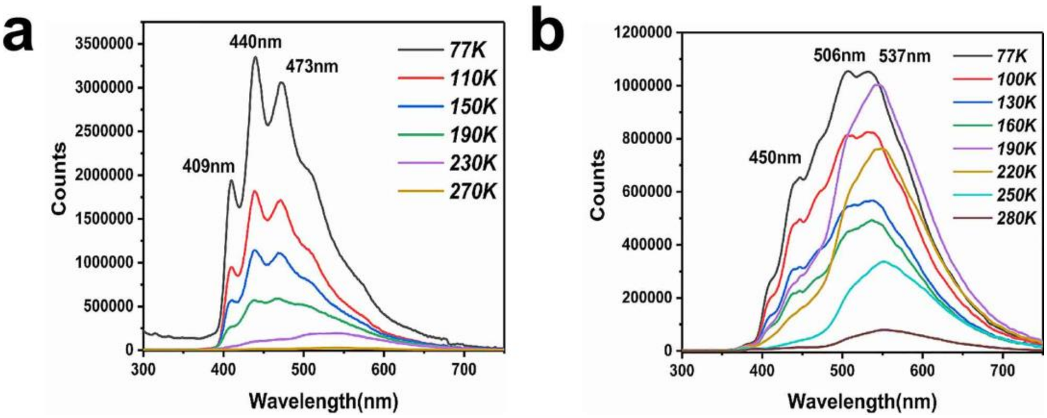

Supplementary Figure 22. Temperature-dependent delayed photoluminescence spectral studies. Temperature-dependent delayed photoluminescence spectra of 2,2-DB doped film with 0.3 mg/mL doping concentration (a) without irradiation and (b) with 45 min irradiation.

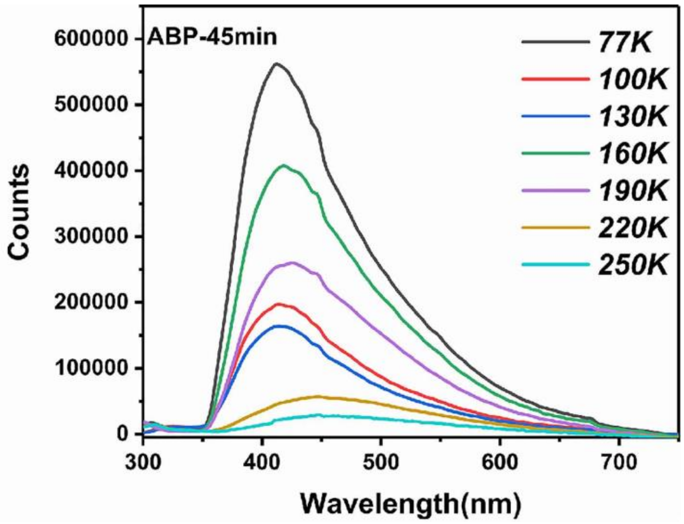

Supplementary Figure 23. Temperature-dependent delayed photoluminescence spectra of ABP-doped film with 0.3 mg/mL doping concentration after 45 min irradiation.

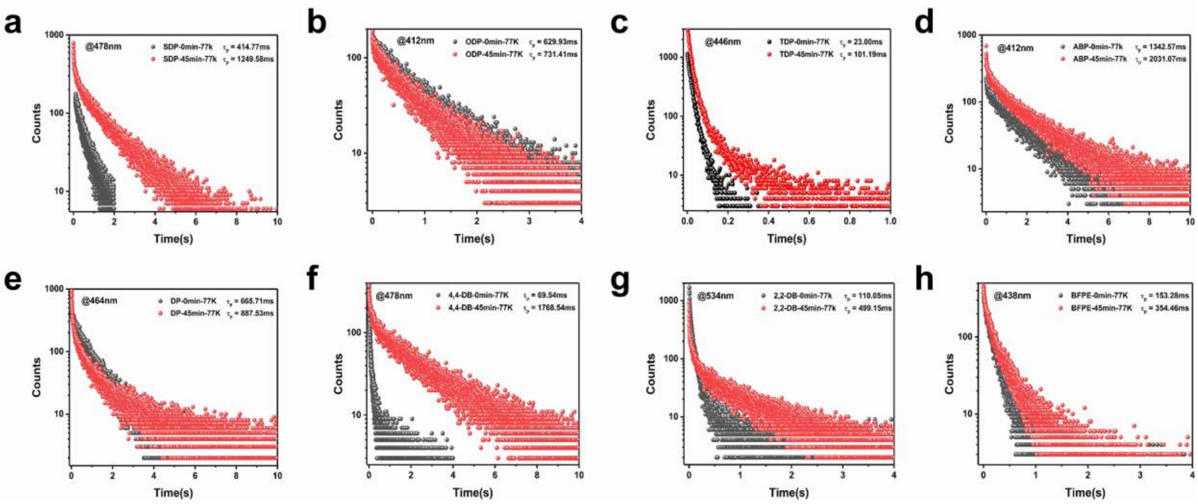

Supplementary Figure 24. Phosphorescence decay studies. Phosphorescence decay curves of (a) SDP, (b) ODP, (c) TDP, (d) DP, (e) 4,4-DB, (f) 2,2-DP, and (g) BFPE doped films at 0.3 mg/mL doping concentration for 0 min and 45 min irradiation at 77 K.

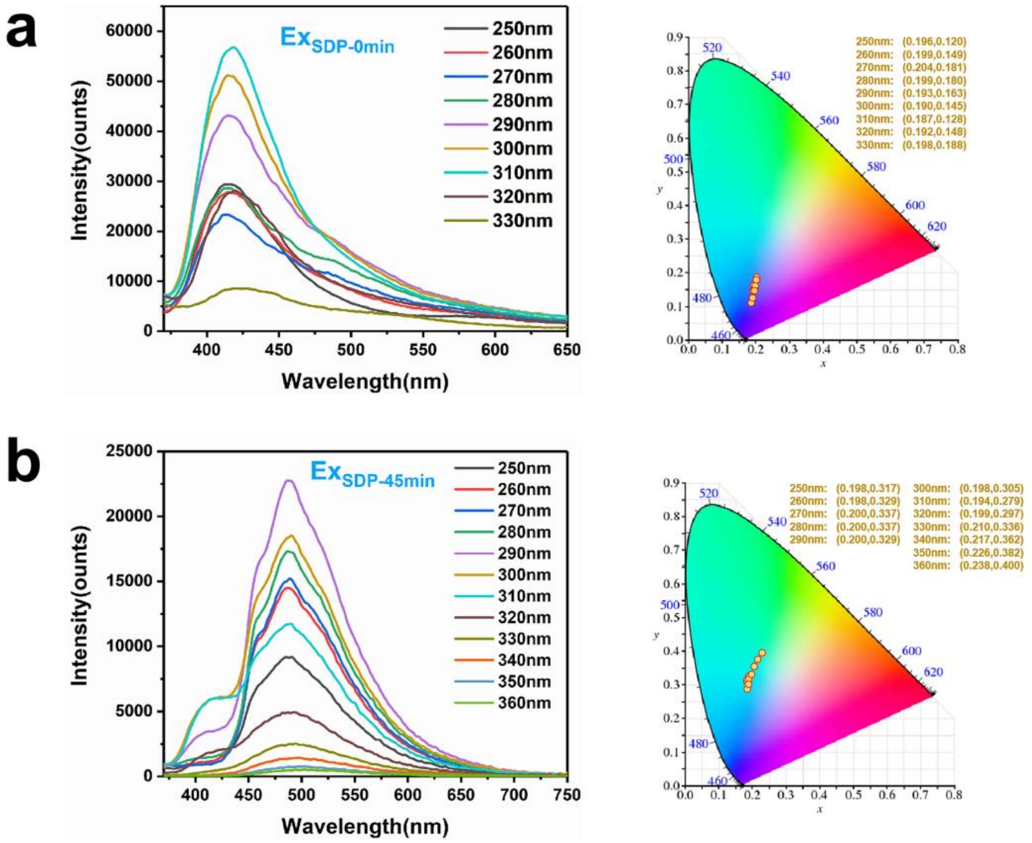

Supplementary Figure 25. Delayed photoluminescence spectral studies. Delayed photoluminescence spectra of SDP doped film at 0.3 mg/mL doping concentration (a) without irradiation and (b) with 45 min irradiation, upon excitation at 250, 260, 270, 280, 290, 300, 320, 330, 340, 350, and 360 nm under ambient conditions, and corresponding CIE coordinates.

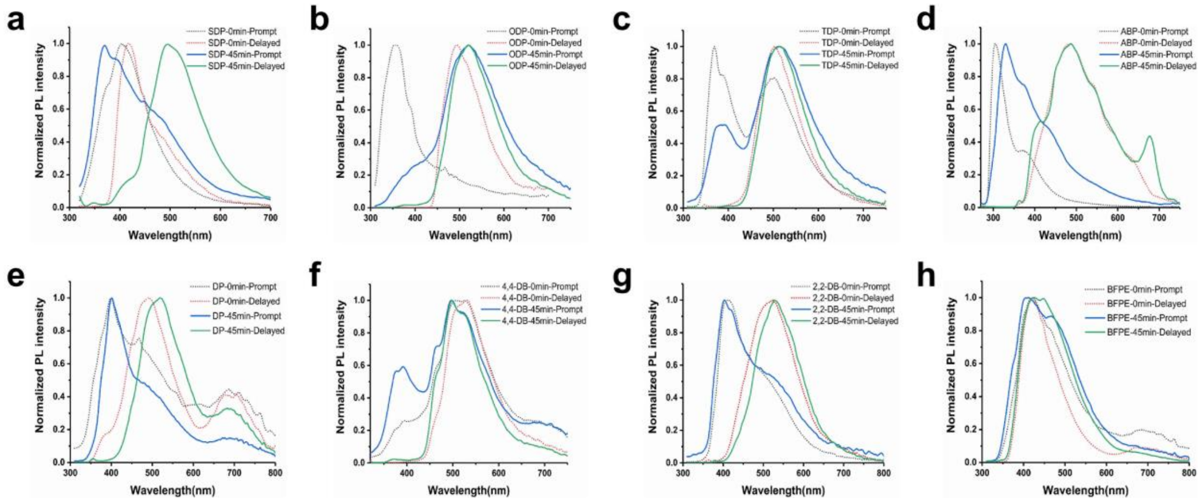

Supplementary Figure 26. Photoluminescence spectral studies. Normalized prompt and 5 ms delayed photoluminescence spectra of (a) SDP, (b) ODP, (c) TDP, (d) ABP, (e) DP, (f) 4,4-DB, (g) 2,2-DP, and (h) BFPE doped films at 0.3 mg/mL doping concentration at room temperature (dash line: without irradiation, solid line: with 45 min irradiation).

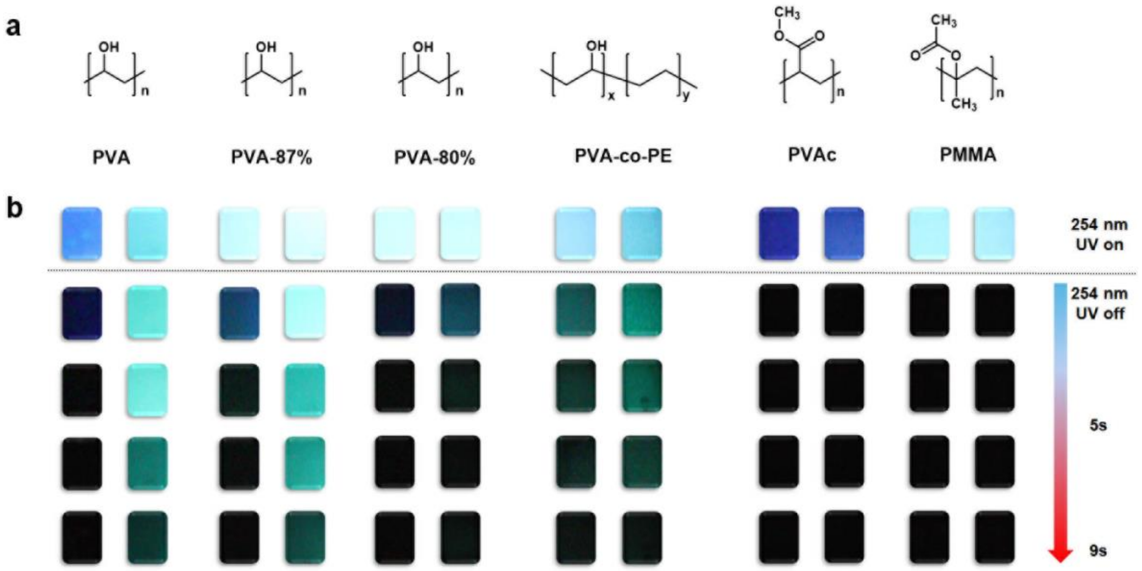

Supplementary Figure 27. Polymer structures and photographs of SDP-doped polymers. (a) Six common polymer matrices including 100% hydrolyzed polyvinyl alcohol (PVA), 87% hydrolyzed PVA (PVA-87%), 80% hydrolyzed PVA (PVA-80%), poly(vinyl alcohol-co-ethylene) (PVA-co-PE), polyvinyl acetate (PVAc), and polymethyl methacrylate (PMMA). (b) Photographs of SDP-doped polymer matrices before and after turning off UV 254 nm light (left: without irradiation; right: with 45 min UV irradiation by UV 254 nm).

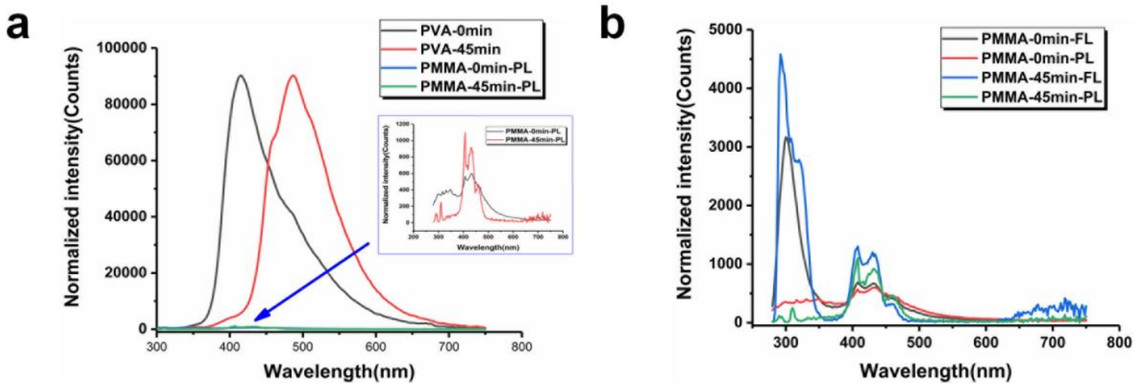

Supplementary Figure 28. Photophysical properties at ambient conditions. (a) Delayed photoluminescence spectra of SDP doped PVA and PMMA, respectively. (b) Normalized prompt photoluminescence spectra of SDP doped PMMA (delayed time: 5 ms).

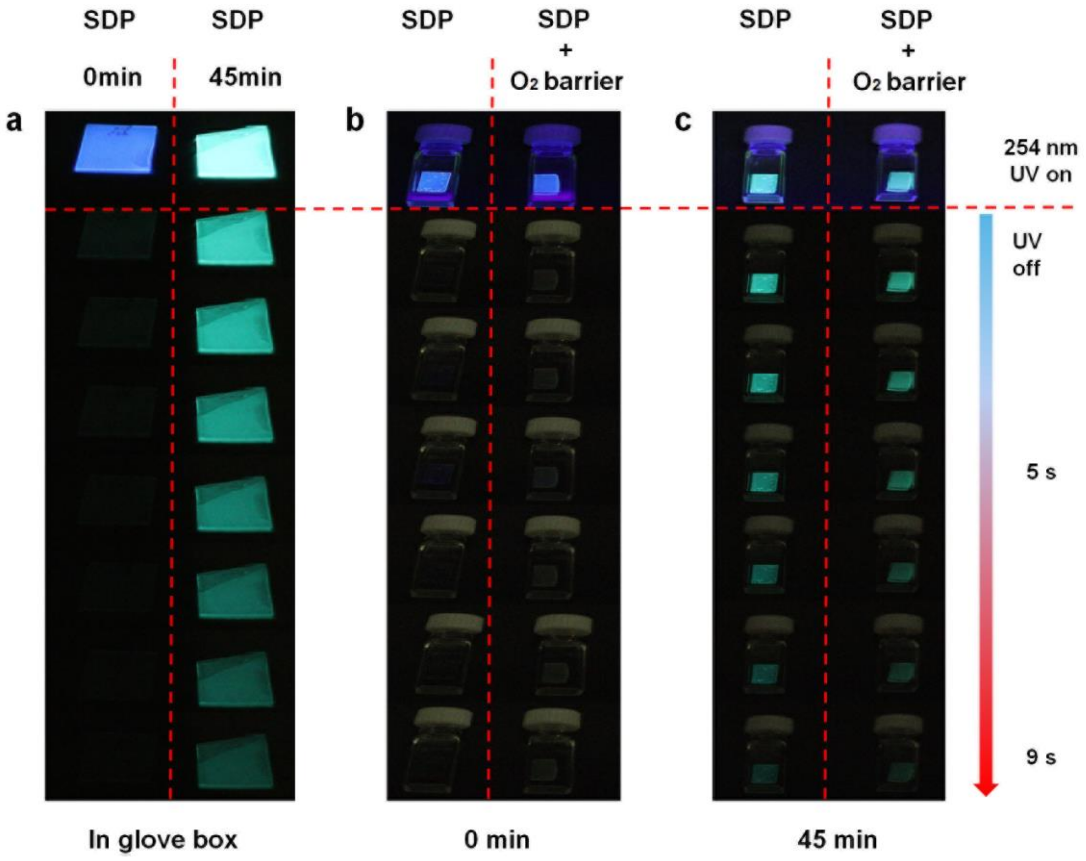

Supplementary Figure 29. Photographs of SDP doped systems before and after turning off UV 254 nm light source. (a) Phosphorescence emission of SDP doped PVA film irradiated at ambient conditions and in a glove box. (b,c) Phosphorescence emission of SDP doped film and SDP doped film sealed with an ethylene-vinyl alcohol copolymer as the oxygen-barrier layer irradiated for 0 min and 45 min, respectively. Cuvette was filled with oxygen.

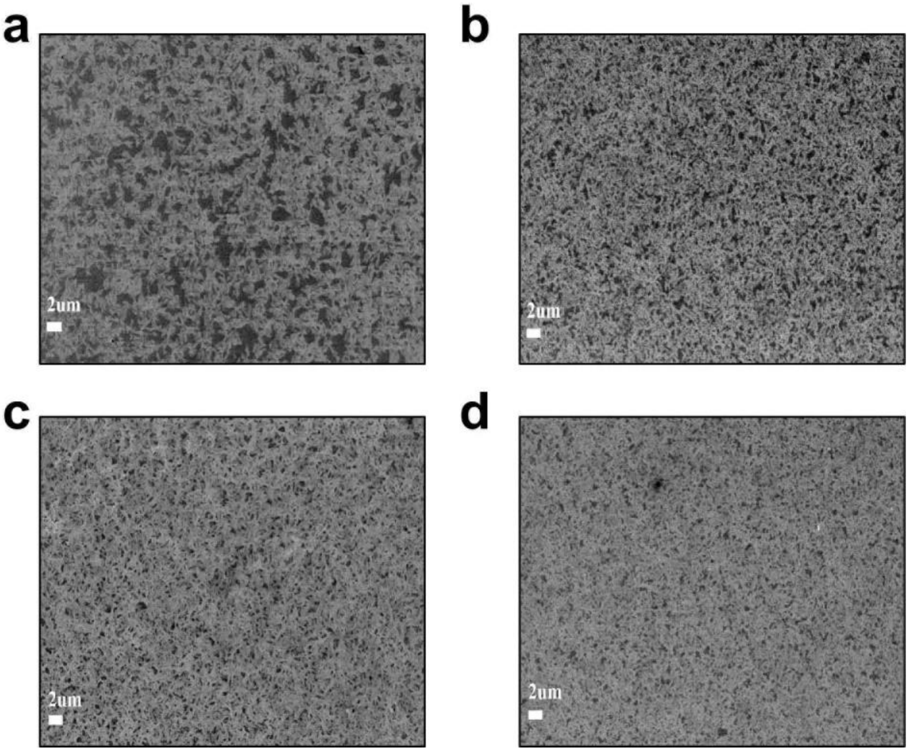

Supplementary Figure 30. SEM images. SEM images (2000 X) of SDP doped film at 0.3 mg/mL doping concentration after irradiation for (a) 0 min, (b) 5 min, (c) 45 min, and (d) 120 min.

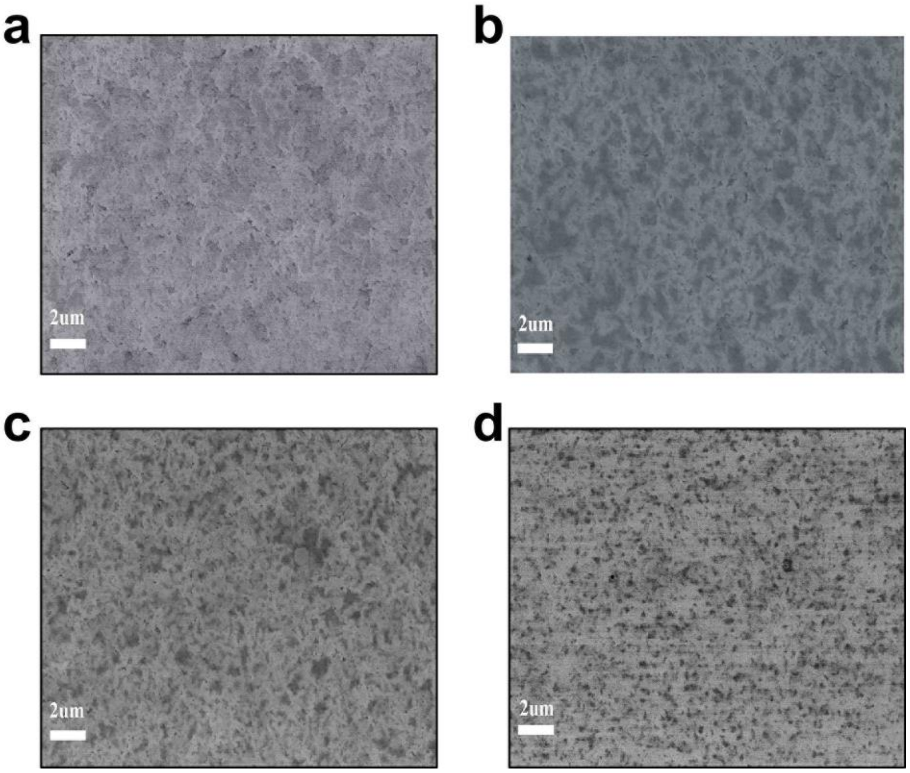

Supplementary Figure 31. SEM images. SEM images (5000 X) of SDP doped film with 0.3 mg/mL doping concentration after irradiation for (a) 0 min, (b) 5 min, (c) 45 min, and (d) 120 min.

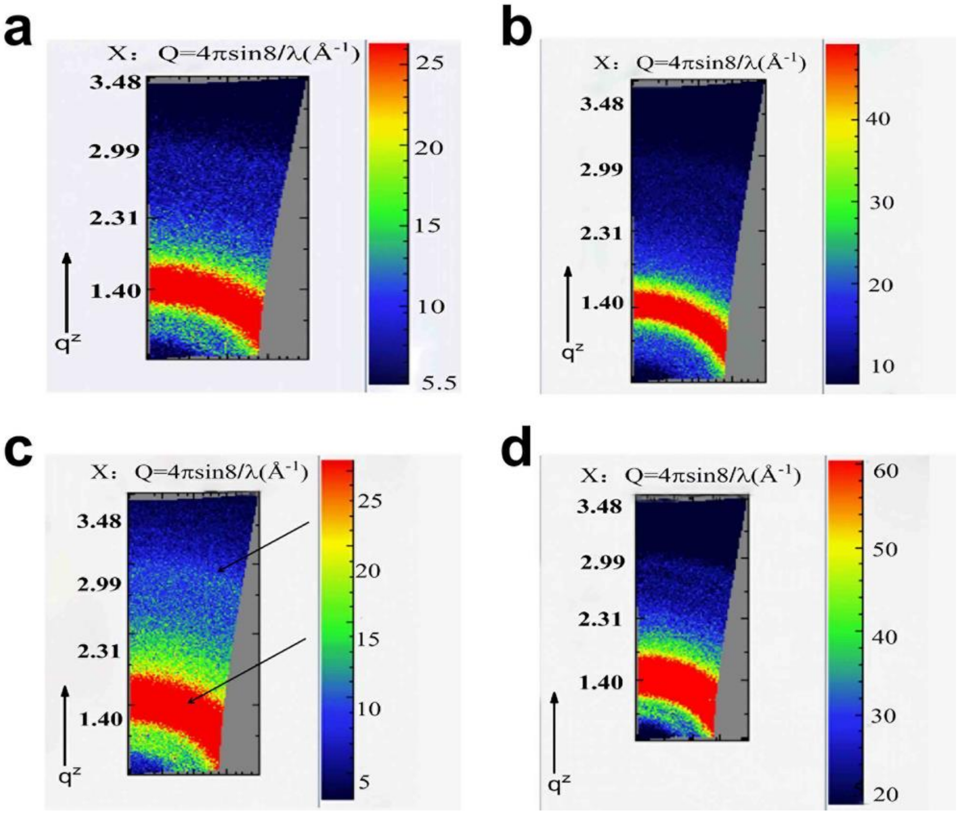

Supplementary Figure 32. 2D GiWAXS out-of-plane patterns. 2D GiWAXS out-of-plane patterns of SDP doped film at 0.3 mg/mL doping concentration after irradiation for (a) 0 min, (b) 5 min, (c) 45 min, and (d) 120 min.

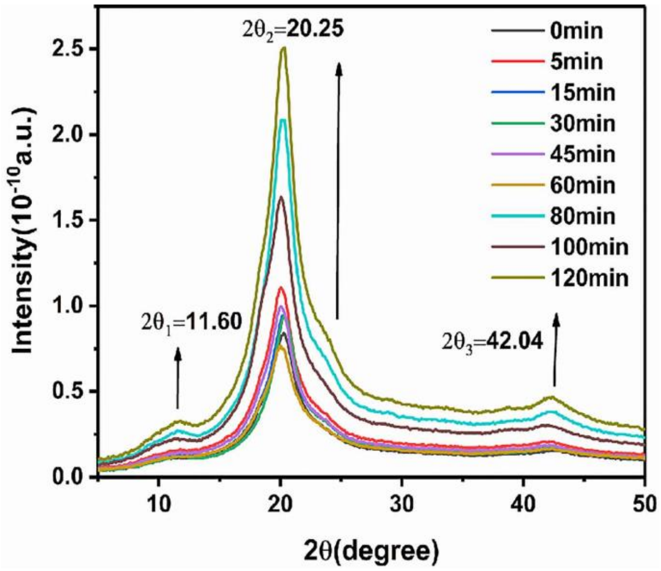

Supplementary Figure 33. 1D scattering profiles in the q_z direction of 2D GiWAXS patterns for SDP doped film at 0.3 mg/mL doping concentration after irradiation from 0 min to 120 min.

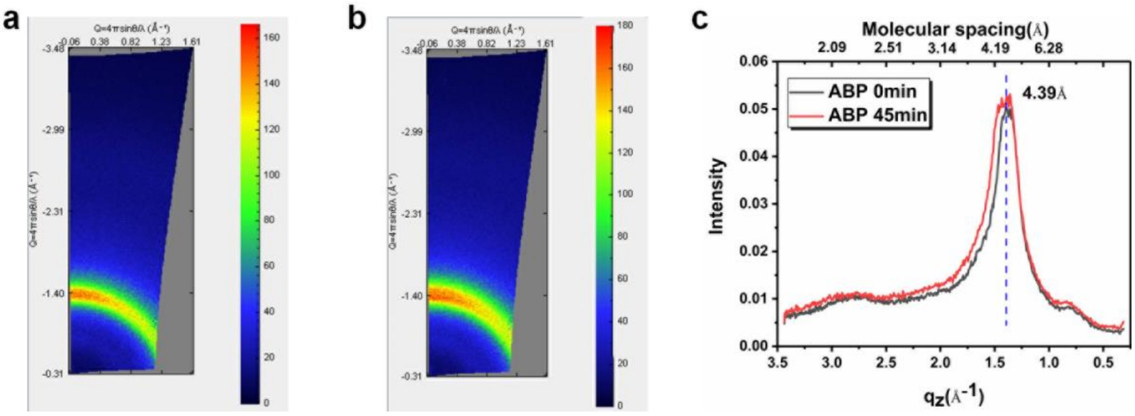

Supplementary Figure 34. 2D GiWAXS out-of-plane patterns. 2D GiWAXS out-of-plane patterns of ABP doped film at 0.3 mg/mL doping concentration after irradiation for (a) 0 min and (b) 45 min. (c) 1D scattering profiles in the q_z direction of ABP doped film for 0 and 45 min.

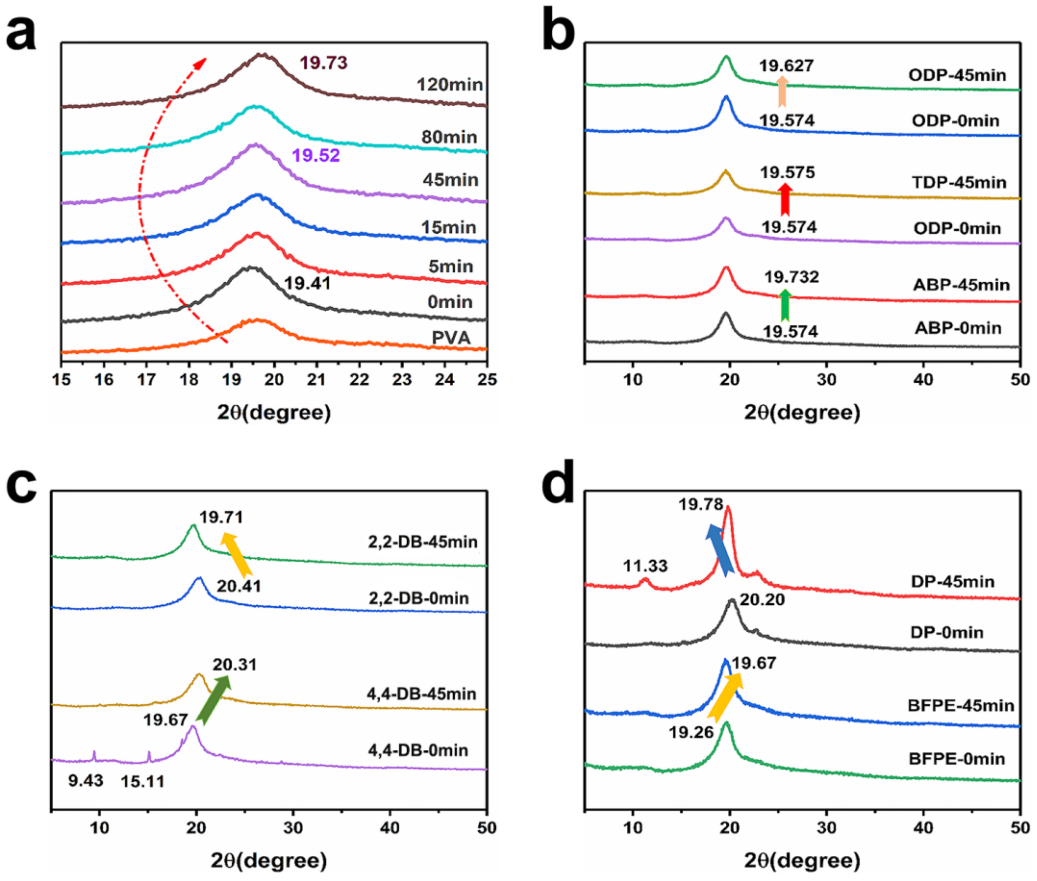

Supplementary Figure 35. Powder XRD patterns. (a-d) Powder XRD patterns of SDP, ODP, TDP, ABP, DP, 4,4-DB, 2,2-DP, and BFPE doped films at 0.3 mg/mL doping concentration with different irradiation times.

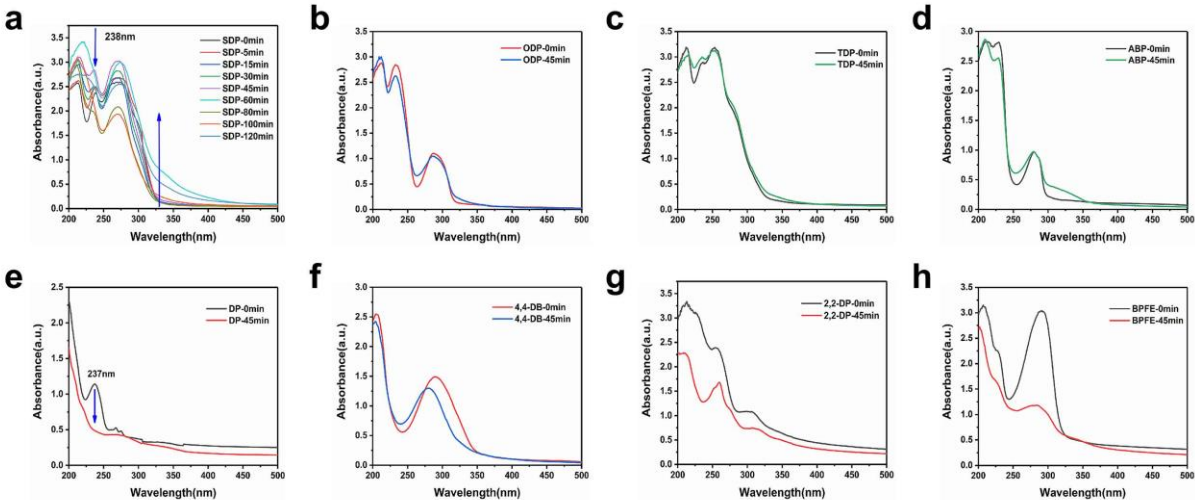

Supplementary Figure 36. UV-vis spectral studies. UV-vis spectra of (a) SDP, (b) ODP, (c) TDP, (d) ABP, (e) DP, (f) 4,4-DB, (g) 2,2-DP, and (h) BFPE doped films at 0.3 mg/mL doping concentration with different irradiation times.

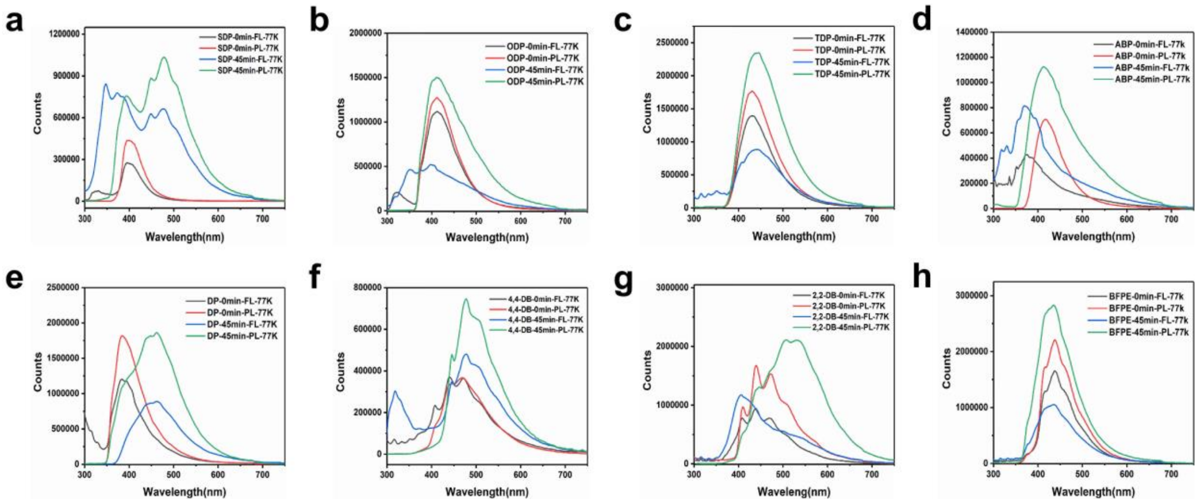

Supplementary Figure 37. Delayed photoluminescence spectral studies. Delayed photoluminescence spectra of (a) SDP, (b) ODP, (c) TDP, (d) ABP, (e) DP, (f) 4,4-DB, (g) 2,2-DP, and (h) BFPE doped films at 0.3 mg/mL doping concentration with 0 min and 45 min irradiation at 77 K.

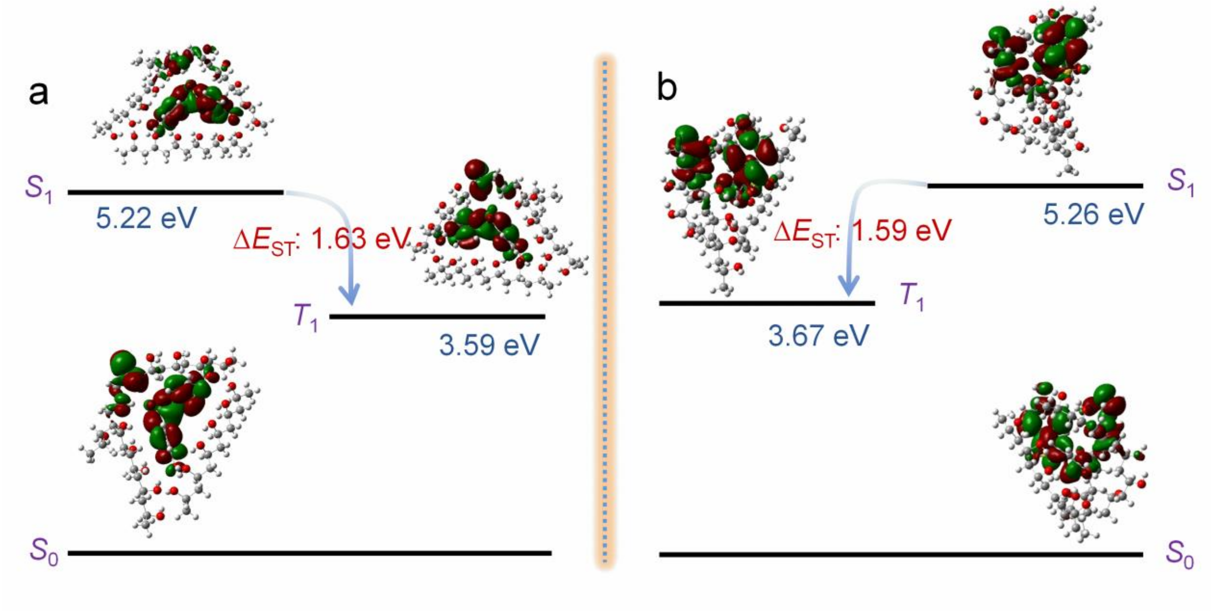

Supplementary Figure 38. DFT calculations for SDP doped PVA film. Molecular orbitals and energy levels of the first singlet excited state (S_1) and the first triplet excited state (T_1) (a) without irradiation and (b) with irradiation.

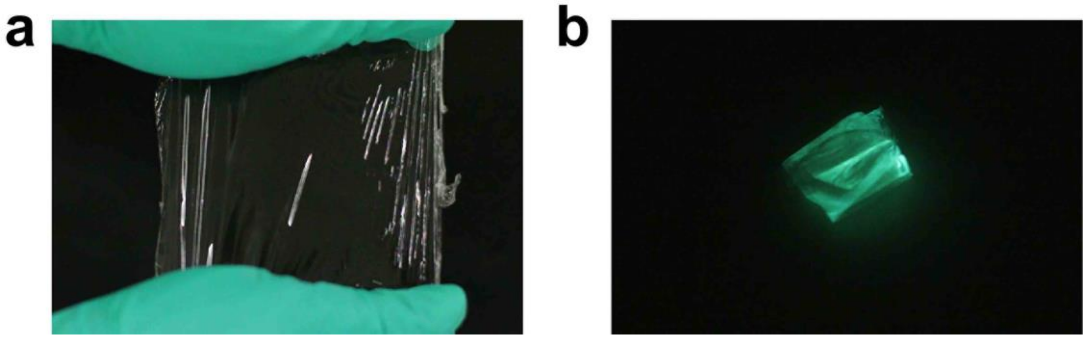

Supplementary Figure 39. Transparent and flexible application studies. (a,b) Transparent and flexible application of SDP doped film at 0.3 mg/mL doping concentration.

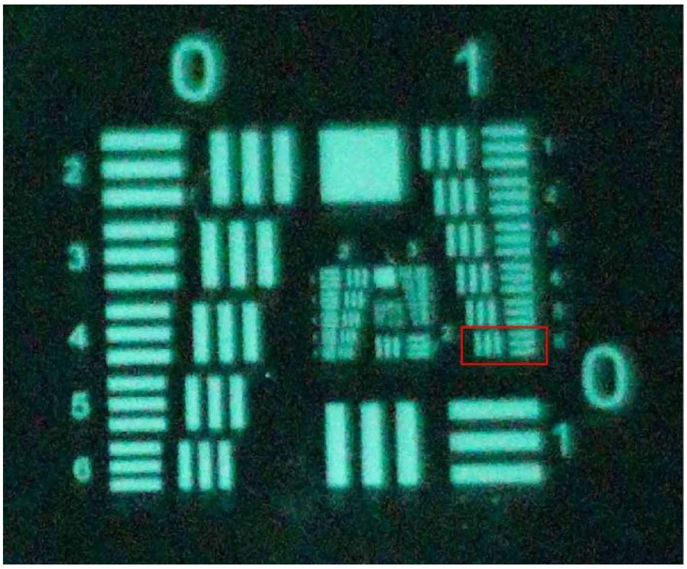

Supplementary Figure 40. USAF 1951 test target and achieved maximum resolution by SDP doped film, showing the line separation in group 1, element 6.

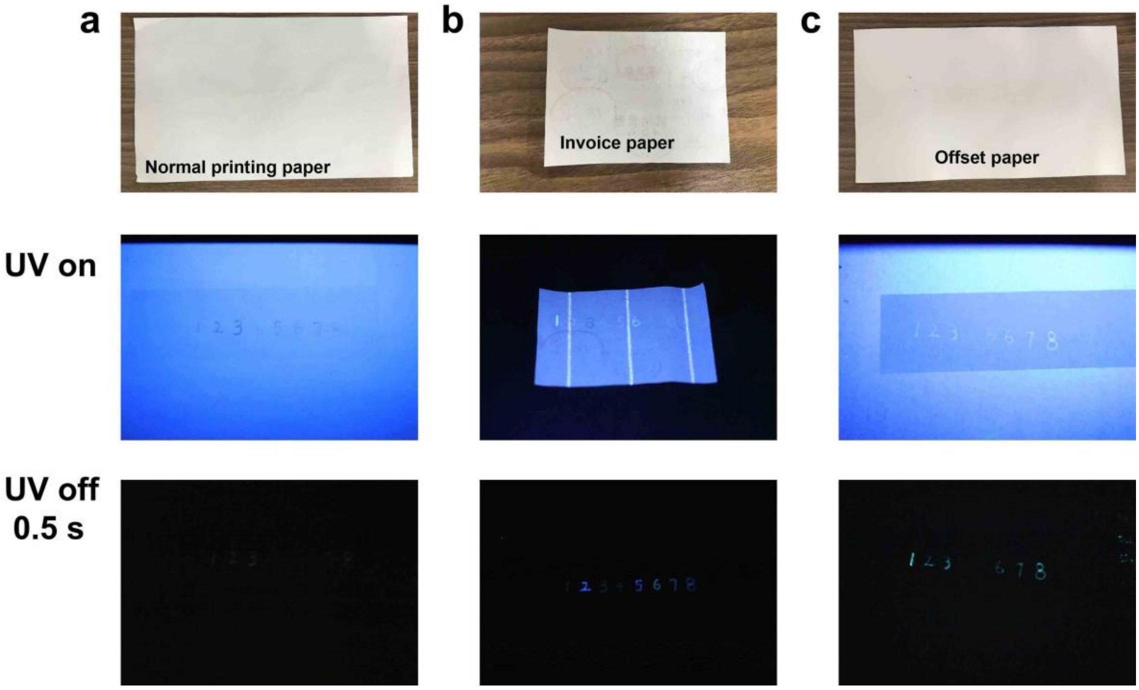

Supplementary Figure 41. Irradiation time-dependent anticounterfeiting photographs (after 0.5 s) of eight phosphor-doped systems as inks under ambient conditions. Numbers 12345678 were encrypted with SDP, ODP, TDP, ABP, DP, 4,4-DB, 2,2-DP, and BFPE doped systems on different substrates, respectively. (a) Normal printing A4 paper. (b) Invoice paper. (c) Offset A4 paper.

Supplementary Table 1. Phosphorescence decay lifetime (τ_{phos}) and phosphorescent quantum yield (Φ_{phos}) at room temperature for SDP, ODP, TDP, DP, 4,4-DB, 2,2-DP, and BFPE doped films at 0.3 mg/mL doping concentration with 0 min and 45 min irradiation.

Systems	SDP	ODP	TDP	ABP	DP	4,4-DB	2,2-DB	BFPE
τ_{phos} (ms) 0 min	58.03	38.54	19.70	26.59	21.52	21.59	168.87	22.13
45 min	828.81	149.66	70.70	25.06	212.73	186.55	257.06	222.09
Φ_{phos} (%) 0min	2.06	0.21	1.13	1.56	4.84	1.75	2.95	0.07
45min	4.96	1.03	1.55	4.38	8.67	1.59	7.35	1.16

Supplementary Table 2. Dynamic photophysical parameters of SDP, ODP, TDP, ABP, DP, 2,2-DB, 4,4-DB, and BFPE doped films at 0.3 mg/mL doping concentration under ambient conditions.

Systems	Irradiation time (min)	Prompt photoluminescence					Delayed photoluminescence			
		τ_{Fluo} (ns)	Φ_{Fluo} (%)	K_r^{Fluo} (s^{-1})	K_{nr}^{Fluo} (s^{-1})	K_{isc} (s^{-1})	τ_{Phos} (ms)	Φ_{Phos} (%)	K_r^{Phos} (s^{-1})	K_{nr}^{Phos} (s^{-1})
SDP	0min	2.76	14.43	4.5×10^7	3.10×10^8	7.46×10^6	58.03	2.06	35.5×10^{-2}	16.88
	5min	3.13	13.23	3.8×10^7	2.77×10^8	4.73×10^6	86.95	1.48	17.0×10^{-2}	11.33
	15min	4.30	8.67	1.6×10^7	2.12×10^8	4.33×10^6	408.36	1.86	4.6×10^{-2}	2.40
	30min	5.90	7.07	0.80×10^7	1.58×10^8	4.02×10^6	628.24	2.37	3.8×10^{-2}	1.55
	45min	8.09	8.74	0.47×10^7	1.13×10^8	6.13×10^6	828.81	4.96	6.0×10^{-2}	1.15
	60min	9.20	6.78	0.19×10^7	1.01×10^8	5.42×10^6	641.25	4.99	7.8×10^{-2}	1.48
	80min	4.18	5.51	0.10×10^7	2.26×10^8	1.22×10^6	596.99	5.09	8.5×10^{-2}	1.59
	100min	4.74	5.34	0.08×10^7	0.20×10^8	1.04×10^6	522.58	4.95	9.4×10^{-2}	1.82
	120min	1.86	4.90	0.03×10^7	5.11×10^8	2.60×10^6	402.29	4.84	12.0×10^{-2}	2.37
ODP	0min	0.95	1.25	1.32×10^7	10.4×10^8	2.21×10^6	38.54	0.21	5.44×10^{-2}	25.89

	45min	0.96	1.74	1.83×10^{7}	10.2×10^{8}	10.8×10^{6}	149.66	1.03	6.89×10^{-2}	6.61
TDP	0min	0.55	2.96	5.38×10^{7}	17.4×10^{8}	20.5×10^{6}	19.70	1.13	5.74×10^{-1}	50.19
	45min	5.13	2.16	0.42×10^{7}	1.88×10^{8}	3.02×10^{6}	70.70	1.55	2.19×10^{-1}	13.93
ABP	0min	2.31	6.48	2.81×10^{7}	3.98×10^{8}	6.75×10^{6}	26.59	1.56	$5.87\times10^{-}$	37.02
	45min	0.41	4.59	11.2×10^{7}	22.2×10^{8}	1.06×10^{8}	25.06	4.38	17.5×10^{-1}	38.16
DP	0min	1.85	18.44	9.97×10^{7}	4.15×10^{8}	26.2×10^{6}	350.06	4.84	1.38×10^{-1}	2.71
	45min	2.04	55.08	27.0×10^{7}	1.78×10^{8}	42.5×10^{6}	480.18	8.67	1.81×10^{-1}	1.90
4,4-DB	0min	1.87	14.02	7.48×10^{7}	4.50×10^{8}	9.36×10^{6}	21.59	1.75	8.11×10^{-1}	45.51
	45min	1.77	12.28	6.94×10^{7}	4.86×10^{8}	8.98×10^{6}	186.55	1.59	0.85×10^{-1}	5.28
2,2-DB	0min	7.66	6.34	0.83×10^{7}	1.20×10^{8}	2.70×10^{6}	168.87	2.07	1.23×10^{-1}	5.80
	45min	13.85	15.43	11.1×10^{7}	0.56×10^{8}	5.31×10^{6}	257.06	7.35	2.86×10^{-1}	3.60
BFPE	0min	0.47	2.98	6.34×10^{7}	20.6×10^{8}	1.49×10^{6}	22.13	0.07	3.16×10^{-2}	45.16
	45min	2.97	15.00	5.03×10^{7}	2.81×10^{8}	3.89×10^{6}	222.09	1.16	5.22×10^{-2}	4.45

Supplementary Table 3. Luminous intensity (mcd/cm^{-1}) of SDP, ODP, TDP, ABP, DP, 4,4-DB, 2,2-DP, and BFPE doped films at 0.3 mg/mL doping concentration for 0 min and 45 min irradiation.

Systems	SDP	ODP	TDP	ABP	DP	4,4-DB	2,2-DB	BFPE
0min	2.93	0.37	-	-	0.63	1.93	1.73	0.08
45min	15.22	9.55	0.01	-	2.31	19.72	3.45	0.43

Supplementary References

1. Frisch, M. J. et al. Gaussian 09, Revision D.01, Gaussian, Inc., Wallingford CT, 2013.

2. Feng, H. et al. Tuning molecular emission of organic emitters from fluorescence to phosphorescence through push-pull electronic effects. *Nat. Commun*. **11**, 2617 (2020).

3. Tao, Y. et al. Resonance-activated spin-flipping for efficient organic ultralong room-temperature phosphorescence. *Adv. Mater*. **30**, 1803856 (2018).

4. Li, M. P. et al. Prolonging ultralong organic phosphorescence lifetime to 2.5 s through confining rotation in molecular rotor. *Adv. Opt. Mater*. **7**, 1800820 (2019).
