# Supplementary material for: Ultraviolet irradiation-responsive dynamic ultralong organic phosphorescence in polymeric systems
Source: Nat Commun. 2021 Apr 16;12:2297. doi: 10.1038/s41467-021-22609-y (PMC8052444; doi:10.1038/s41467-021-22609-y)
Supplement: Supplementary file 3 — Description of Additional Supplementary Files [file 41467_2021_22609_MOESM3_ESM.pdf]

Description of Additional Supplementary Files

File Name: Supplementary Movie 1
Description: SDP, ODP, TDP, DP, 4,4-DB, 2,2-DP, and BFPE doped films at 0.3 mg/mL doping concentration under UV-254 nm lamp on and off.

File Name: Supplementary Movie 2
Description: Screen printing of SDP doped film at 0.3 mg/mL doping concentration on a glass substrate under UV-254 nm lamp on and off. After switching off the UV lamp, a green afterglow was observed by the naked eye.

File Name: Supplementary Movie 3
Description: Screen printing of 2,2-DB doped film at 0.3 mg/mL doping concentration on a glass substrate under UV-254 nm lamp on and off. After switching off the UV lamp, a yellow afterglow was clearly observed by the naked eye.
